# β_3_ Integrin and Src facilitate transforming growth factor-β mediated induction of epithelial-mesenchymal transition in mammary epithelial cells

**DOI:** 10.1186/bcr1524

**Published:** 2006-07-19

**Authors:** Amy J Galliher, William P Schiemann

**Affiliations:** 1 UCHSC, Fitzsimons Campus, Department of Pharmacology, Mail Stop 8303, RC1 South Tower, Rm L18-6110, PO Box 6511, Aurora, CO 80045

## Abstract

**Introduction:**

Transforming growth factor (TGF)-β suppresses breast cancer formation by preventing cell cycle progression in mammary epithelial cells (MECs). During the course of mammary tumorigenesis, genetic and epigenetic changes negate the cytostatic actions of TGF-β, thus enabling TGF-β to promote the acquisition and development of metastatic phenotypes. The molecular mechanisms underlying this conversion of TGF-β function remain poorly understood but may involve signaling inputs from integrins.

**Methods:**

β_3 _Integrin expression or function in MECs was manipulated by retroviral transduction of active or inactive β_3 _integrins, or by transient transfection of small interfering RNA (siRNA) against β_3 _integrin. Altered proliferation, invasion, and epithelial-mesenchymal transition (EMT) stimulated by TGF-β in control and β_3 _integrin manipulated MECs was determined. Src involvement in β_3 _integrin mediated alterations in TGF-β signaling was assessed by performing Src protein kinase assays, and by interdicting Src function pharmacologically and genetically.

**Results:**

TGF-β stimulation induced α_v_β_3 _integrin expression in a manner that coincided with EMT in MECs. Introduction of siRNA against β_3 _integrin blocked its induction by TGF-β and prevented TGF-β stimulation of EMT in MECs. β_3 _integrin interacted physically with the TGF-β receptor (TβR) type II, thereby enhancing TGF-β stimulation of mitogen-activated protein kinases (MAPKs), and of Smad2/3-mediated gene transcription in MECs. Formation of β_3 _integrin:TβR-II complexes blocked TGF-β mediated growth arrest and increased TGF-β mediated invasion and EMT. Dual β_3 _integrin:TβR-II activation induced tyrosine phosphorylation of TβR-II, a phosphotransferase reaction mediated by Src *in vitro*. Inhibiting Src activity in MECs prevented the ability of β_3 _integrin to induce TβR-II tyrosine phosphorylation, MAPK activation, and EMT stimulated by TGF-β. Lastly, wild-type and D119A β_3 _integrin expression enhanced and abolished, respectively, TGF-β stimulation of invasion in human breast cancer cells.

**Conclusion:**

We show that β_3 _integrin alters TGF-β signaling in MECs via Src-mediated TβR-II tyrosine phosphorylation, which significantly enhanced the ability of TGF-β to induce EMT and invasion. Our findings suggest that β_3 _integrin interdiction strategies may represent an innovative approach to re-establishing TGF-β mediated tumor suppression in progressing human breast cancers.

## Introduction

Transforming growth factor (TGF)-β is a powerful tumor suppressor that prevents the uncontrolled proliferation of epithelial, endothelial, and hematopoietic cells. In doing so, TGF-β initiates transmembrane signaling by activating its type I and type II serine/threonine kinase receptors (TGF-β receptor TβR-I and TβR-II, respectively). Following its transphosphorylation and stimulation by TβR-II, TβR-I then binds, phosphorylates, and activates the intracellular effectors Smad2 and Smad3, which subsequently complex with Smad4 and translocate to the nucleus to regulate target gene transcription [1]. Although the Smad pathway is by far the most characterized TGF-β activated pathway, TGF-β also governs cell physiology through activation of mitogen-activated protein kinases (MAPKs; including extracellular signal-regulated kinase [ERK]1/2, p38, and c-Jun amino-terminal kinase) and of phosphoinositol-3 kinase (PI3K) [2-5]. Aberrant activation of MAPKs and PI3K often is associated with cancer development in humans. Precisely how TGF-β activates these alternative pathways and how these signals are integrated into the biology and pathology of TGF-β remain to be elucidated fully [6].

TGF-β plays a dual role during mammary tumorigenesis [7-10]. For instance, TGF-β normally prohibits mammary epithelial cell (MEC) cell cycle progression, and consequently suppresses MEC tumorigenesis. However, during the course of mammary tumorigenesis, TGF-β signaling becomes dysregulated and uncoupled from regulation of cell cycle progression. More importantly, altered TGF-β signaling actively contributes to the acquisition and development of metastatic phenotypes, in part through its ability to stimulate epithelial-mesenchymal transitions (EMTs) in cancerous MECs [11]. Indeed, recent evidence suggests that TGF-β suppresses tumorigenesis largely via Smad2/3-mediated growth arrest [1,6,8,12,13], whereas its ability to promote tumorigenesis and EMT occurs via the integration of Smad2/3 signals with those arising in response to activation of RhoA, MAPKs (e.g. ERK1/2 and p38 MAPK), and PI3K pathways [2,3,14-16]. Thus, breast cancer cells have developed effective strategies for circumventing the tumor suppressing activities of TGF-β, while simultaneously selecting for, or even enhancing, its tumor promoting activities [8,17].

Apart from playing a prominent role in preventing cell cycle progression, TGF-β also is a major regulator of cell microenvironments and extracellular matrix (ECM) remodeling. TGF-β alters cell microenvironments in part through its ability to induce the expression of unique subsets of integrins as well as that of their ECM ligands [18]. In doing so, TGF-β enables malignant cells to undergo EMT and, consequently, to escape their tissue of origin [11].

Integrins are heterodimeric transmembrane receptors that bind ECM ligands and couple cells to their microenvironments [19]. Most integrins initiate transmembrane signaling by activating focal adhesion kinase (FAK) and Src family kinases at adhesive sites. FAK and Src further recruit and activate various downstream effectors, such as PI3K and members of the Ras and Rho families of small GTPases [20-22]. Integrins also interact with and couple to receptor tyrosine kinases (RTKs) to promote cell survival, proliferation, and migration in response to soluble growth factors and cytokines [23,24]. One such integrin, namely α_v_β_3_, binds to arginine-glycine-aspartic acid (RGD) amino acid containing components of the ECM, such as vitronectin, fibronectin, and osteopontin, and mediates RTK activation of MAPKs and cell invasion [23-25]. Similar to the effects of tumorigenesis on TGF-β signaling, integrin expression is altered during tumorigenesis, including developing tumors of the breast [26,27]. In particular, altered α_v_β_3 _integrin expression correlates with mammary tumorigenesis, particularly the processes of breast cancer cell invasion and metastasis [25,27-31], raising the possibility that differential integrin expression may contribute to the tumor promoting activities of TGF-β. Indeed, TGF-β stimulated EMT is abrogated by treatments that inhibit MEC integrin adhesion, suggesting a need for integrins in mediating TGF-β signaling [32,33].

To further investigate the role of altered integrin expression in regulating the MEC response to TGF-β, we determined the effects of β_3 _integrin expression on the ability of TGF-β to regulate NMuMG (normal murine mammary gland) cell proliferation, invasion, and EMT. We found that treatment of NMuMG cells with TGF-β induced their expression of α_v_β_3 _integrin, an event that coincided with TGF-β stimulation of EMT. Accordingly, β_3 _integrin deficiency abolished the ability of TGF-β to induce EMT in MECs. Moreover, we found that β_3 _integrin interacted physically with TβR-II at the cell surface, leading to conversion of TGF-β from a suppressor of NMuMG cell growth to a promoter of their invasiveness and EMT. Mechanistically, activated β_3 _integrin recruited Src to β_3 _integrin:TβR-II complexes, where it tyrosine phosphorylated TβR-II, leading to enhanced activation of MAPKs and induction of EMT stimulated by TGF-β. Importantly, abolishing Src activity or expression in NMuMG cells prevented β_3 _integrin-mediated tyrosine phosphorylation of TβR-II and, consequently, EMT stimulated by TGF-β. Finally, we found that the acquisition of a metastatic phenotype in MCF10A derivatives, which serve as a model of human breast cancer progression regulated by TGF-β [9], coincided with upregulated β_3 _integrin and FAK expression. Similar to its effects in NMuMG cells, β_3 _integrin expression significantly enhanced TGF-β mediated stimulation of cell invasion in benign MCF10A cells as well as in their highly metastatic counterparts MCF10CA1a cells. Importantly, the expression of D119A β_3 _integrin in these metastatic cells completely abolished the ability of TGF-β to induce MCF10CA1a cell invasion.

Taken together, our findings identify a novel convergence point in MECs that enables β_3 _integrins to override the tumor suppressing activities of TGF-β, suggesting that integrin interdiction strategies may one day represent an innovative approach to re-establishing TGF-β mediated tumor suppression in progressing human breast cancers.

## Materials and methods

### Retroviral plasmids and expression

The cDNAs encoding wild-type (WT) human β_3 _integrin, as well as its inactive mutant D119A (D→A point mutation at position 119), were generously provided by Dr Mark H Ginsberg (University of California at San Diego, San Diego, CA, USA [34]). Retroviral β_3 _integrin vectors were synthesized by PCR amplification using oligonucleotides containing *Bgl*II (amino terminus) and *Xho*I (carboxyl terminus) restriction sites, and subsequently ligated into identical sites immediately upstream of the IRES in the bicistronic retroviral vector pMSCV-IRES-GFP (plasmid murine stem cell virus-internal ribosomal entry site-green fluorescent protein) or pMSCV-IRES-YFP (yellow fluorescent protein). [35] All β_3 _integrin inserts were sequenced in their entirety on an Applied Biosystems 377A DNA sequencing machine (Applied Biosystems, Foster City, CA USA).

Full-length human c-Src cDNA was PCR amplified from IMAGE clone 4871614 (Invitrogen, Carlsbad, CA, USA) using oligonucleotides containing *Hind*III (amino terminus) and *Xba*I (carboxyl terminus) restriction sites, respectively. The resulting PCR product was ligated into corresponding sites in pcDNA3.1/Myc-His B vector (Invitrogen) to carboxyl-terminally tag c-Src with a Myc and (His)_6_-tag. Kinase dead (Lys295Met) or constitutively active (Tyr530Phe) c-Src mutants were generated by site-directed mutagenesis using the QuikChange site-directed mutagenesis kit (Stratagene, La Jolla, CA, USA). Afterward, Myc-His tagged WT and mutant c-Src cDNAs were amplified by PCR and ligated into *Eco*RI (amino terminus) and *Bgl*II (carboxyl terminus) restriction sites in pMSCV-IRES-GFP. All c-Src inserts sequenced in their entirety on an Applied Biosystems 377A DNA sequencing machine.

NMuMG cells were cultured in Dulbecco's modified Eagle's medium supplemented with 10% fetal bovine serum (FBS) and 10 μg/ml insulin. MCF10A and MCF10CA1a cells were cultured as previously described [9]. Stable expression of individual β_3 _integrin subunits or c-Src derivatives in NMuMG and MCF10A cells was accomplished by their overnight infection with control (i.e. pMSCV-IRES-GFP or pMSCV-IRES-YFP), WT or D119A β_3 _integrin, or mutant c-Src retroviral supernatants produced by EcoPac2 retroviral packaging cells (Clontech, San Diego, CA, USA), as described previously [36]. Cells expressing GFP, YFP, or both fluorescent proteins were isolated and collected 48 hours later on a MoFlo cell sorter (Cytomation, Fort Collins, CO, USA), and subsequently were expanded to yield stable polyclonal populations of control (i.e. GFP or YFP), β_3 _integrin (i.e. WT or D119A), or mutant c-Src (i.e. kinase-dead or constitutively active) expressing cells. Expression of recombinant β_3 _integrins in individual NMuMG cell lines was monitored by immunoblotting detergent solubilized whole cell extracts with antibodies against the extracellular domain of β_3 _integrin (Santa Cruz Biotechnology, Santa Cruz, CA, USA), whereas expression of mutant c-Src protein kinases was detected by immunoblotting with either anti-Src (1:1000 dilution; Santa Cruz Biotechnology) or anti-Myc (1:1000 dilution; Covance, Princeton, New Jersey USA) antibodies.

### Fluorescence-activated cell sorting analysis

Control (GFP), WT, or D119A β_3 _integrin expressing NMuMG cells were cultured in the absence or presence of TGF-β_1 _(5 ng/ml) for 36 hours to stimulate EMT. Afterward, 1 × 10^6 ^cells were trypsinized, washed, and incubated in fluorescence-activated cell sorter (FACS) buffer (1% bovine serum albumen in phosphate-buffered saline [PBS]) supplemented with a 1:20 dilution of either PE-conjugated anti-mouse α_v _integrin (BD Pharmingen, San Diego, CA, USA) or PE-conjugated anti-human β_3 _integrin antibodies (BD Pharmingen). After a 30 min incubation on ice, the cells were washed twice in PBS and immediately fixed with 1% paraformaldehyde before fluorescence-activated cell sorting (FACS) analysis of α_v _or β_3 _expression in GFP-positive NMuMG cells.

### Immunofluorescence studies

The ability of TGF-β to alter actin cytoskeletal architecture was monitored essentially as described previously [37,38]. Briefly, control or β_3 _integrin expressing NMuMG cells (30,000 cells/well) were plated onto gelatin coated (0.1% in PBS) glass coverslips in 24-well plates. The cells were stimulated with TGF-β_1 _(5 ng/ml) for 0–36 hours at 37°C. In some experiments, control or β_3 _integrin-expressing NMuMG cells were stimulated with TGF-β_1 _in the absence or presence of the Src kinase inhibitor PP2 (10 μmol/l; Calbiochem, Temecula, CA, USA) or its inactive counterpart PP3 (10 μmol/l; Calbiochem). Upon completion of agonist stimulation, the cells were washed in PBS, fixed in 4% paraformaldehyde, and permeabilized by Triton X-100 (0.2% in PBS). The cells were then blocked in PBS supplemented with 1.5% FBS, followed by incubation with TRITC-phalloidin or FITC-phalloidin (0.25 μmol/l). For α_v_β_3 _integrin staining, the cells were blocked in goat β-globulin (200 μg/ml; Jackson Immunoresearch, West Grove, PA, USA) before sequential incubations with anti-α_v_β_3 _LM609 antibody (1:250 dilution; BD Biosciences, San Jose, CA USA), followed by biotinylated goat anti-mouse antibody (5 μg/ml; Jackson Immunoresearch) and finally by Alexa-streptavidin (1.2 μg/ml; Invitrogen). All images were captured on a Nikon Diaphot microscope.

### RNA interference studies

NMuMG cells lacking either β_3 _integrin or c-Src were generated using SMARTpool small interfering RNAs (siRNAs), in accordance with the manufacturer's recommendations (Dharmacon, Lafayette, CO, USA). Briefly, NMuMG cells (30,000 cells/well) were plated either onto plastic or gelatin coated (0.1% in PBS) glass coverslips in 24-well plates and cultured overnight in antibiotic-free media. Fresh media was added the following morning and the cells were transiently transfected with DharmaFECT One reagent (Dharmacon) supplemented with β_3 _integrin or c-Src siRNAs (100 nmol/l). Thirty-six hours after transfection, the cells were treated with TGF-β_1 _(5 ng/ml) for varying times at 37°C. Upon completion of agonist stimulation, the cells were harvested and prepared for immunoblotting and immunofluorescence analyses as above. NMuMG cell transfection efficiency was monitored by co-transfection with si*GLO *RISC-Free siRNA (Dharmacon), which was visualized under fluorescent microscope.

### Cellular phosphorylation assays

Control or β_3 _integrin-expressing NMuMG cells were cultured onto 24-well plates (30,000 cells/well) and allowed to adhere overnight. The next morning, the cells were washed twice in PBS and placed in Dulbecco's modified Eagle's medium supplemented with 0.5% FBS for 4 hours before stimulation with TGF-β_1 _(5 ng/ml) for 0–120 min. In some experiments, the cells were stimulated with Prolactin (100 ng/ml; generously provided by National Hormone and Peptide Program, US National Institutes of Health), 4α-phorbol 12-myristate 13-acetate (PMA; 10 ng/ml; Sigma, St. Louis, MO, USA), or epidermal growth factor (EGF; 100 ng/ml; Upstate, Charlottesville, VA). Afterward, the cells were washed in ice-cold PBS and lysed in 200 μl of buffer H/1% Triton X-100 [36]. Detergent solubilized whole cell extracts were prepared, clarified by microcentrifugation, and subsequently concentrated by acetone precipitation. Recovered proteins were fractionated through 10% SDS-PAGE gels, immobilized electrophoretically to nitrocellulose membranes, and subsequently probed with a 1:250 dilution of either anti-phospho-Smad2, -ERK1/2, or -p38 MAPK polyclonal antibodies (Cell Signaling Technology, Beverly, MA, USA). The resulting immunocomplexes were visualized by enhanced chemiluminescence. Differences in protein loading could not be readily monitored by b-actin immunoreactivity because stable β_3 _integrin expression and TGF-β stimulation significantly elevated b-actin expression in MECs (data not shown [5,32,39]). Thus, differences in protein loading were instead monitored by reprobing stripped membranes with anti-ERK1/2 antibodies (1:2500 dilution; Upstate, Charlottesville, VA USA), whose expression in MECs was unaltered by TGF-β treatment [5,32,39].

### Cell biological assays

The effect of WT and D119A β_3 _integrin expression on various TGF-β stimulated activities in MECs was determined as follows: cell proliferation using 10,000 cells/well in a [^3^H]thymidine incorporation assay, as described elsewhere [35]; cell invasion induced by 10% serum using 350,000 cells/well in a modified Boyden-chamber coated with Matrigel matrices (diluted 1:25 in serum-free Dulbecco's modified Eagle's medium), as described elsewhere [35,40]; and gene expression using 30,000 cells/well in a synthetic pSBE-luciferase reporter gene assay, as described previously [35].

### Iodinated transforming growth factor-β_1 _radioligand binding and cross-linking assay

Control and β_3 _integrin-expressing NMuMG cells were plated onto 10 cm plates and grown until they reached 90% confluency. The radioligand binding and cross-linking of [^125^I]TGF-β_1 _(200 pmol/l) to NMuMG cells was performed as described previously [41]. Afterward, cytokine:receptor complexes contained in detergent-solubilized whole cell extracts were isolated by immunoprecipitation with anti-TβR-II antibodies, as described elsewhere [41]. Immunocomplexes were subsequently fractionated through 7.5% SDS-PAGE and then immobilized electrophoretically to nitrocellulose and probed with anti-β_3 _integrin antibodies. Iodinated TGF-β_1 _bound to cell surface TβR-I and TβR-II was visualized by exposure of the dried nitrocellulose membranes to a phosphor screen, which was developed 1–3 days later on a Molecular Dynamics Typhoon Scanner (GE Healthcare Bio-Sciences Corp., Piscataway, NJ USA).

### Integrin:TβR-II co-immunoprecipitation assays

Control (2.5 × 10^6^) or β_3 _integrin (7 × 10^5^) expressing NMuMG cells were cultured onto 10 cm plates and subsequently stimulated with TGF-β_1 _(5 ng/ml) for varying times in the absence or presence of the Src inhibitor PP2 (10 μmol/l). In some cases, NMuMG cells were held in suspension and replated onto culture dishes previously coated with vitronectin (1 ng/ml; Chemicon, Temecula, CA, USA). Following agonist stimulation, the cells were washed twice in ice-cold PBS and disrupted in Nonidet P-40 lysis buffer [42] (Nonidet P-40; Sigma). The resulting detergent-solubilized whole cell extracts were clarified by microcentrifugation and subjected to the following immunoprecipitation conditions: anti-β_1 _integrin antibodies (Santa Cruz Biotechnology) using 1 mg whole cell extract; anti-β_3 _integrin antibodies using 1 mg whole cell extract; anti-phosphotyrosine 4G10 antibodies (Upstate) using 1 mg whole cell extract; or anti-TβR-I or -TβR-II antibodies using 2 mg whole cell extract as described previously [43]. All immunoprecipitations were incubated for 16 hours at 4°C with slow rotation. The resulting immunocomplexes were collected by microcentrifugation, washed, fractionated through 10% SDS-PAGE gels, and transferred electrophoretically to nitrocellulose membranes, which subsequently were probed with anti-β_3 _integrin (1:1000), anti-TβR-II (1:1500; Santa Cruz Biotechnology), or anti-phosphotyrosine 4G10 (1:1500; Upstate) antibodies.

### *In vitro *tyrosine kinase phosphorylation assays

Tyrosine phosphorylation of TβR-II was determined using an *in vitro *protein kinase assay that measured the ability of either active FAK or Src to phosphorylate recombinant glutathione *S*-transferase (GST)-TβR-II, which contained only the cytoplasmic domain (i.e. the carboxyl terminal 380 amino acids) of TβR-II fused to GST. Protein kinase reactions were performed in a final volume of 30 μl, consisting of 1 μg of GST-TβR-II with either 0.2 μg of FAK or 1 Unit of Src (Cell Signaling Technology). Reactions were initiated by addition of 10 μl of 4× assay buffer [44] and were incubated for 30 min at 30°C. Afterward, the reactions were stopped by addition of 4× sample buffer [44] and boiled for 5 min. Reactions were diluted by addition of 1 ml buffer H/Triton X-100 and immunoprecipitated overnight with anti-TβR-II antibodies. The resulting immunocomplexes were collected, washed, and resuspended in 1X sample buffer. GST fusion proteins were fractionated through 10% SDS-PAGE before their immobilization to nitrocellulose membranes. Tyrosine phosphorylation of GST-TβR-II was visualized by immunoblotting nitrocellulose membranes with anti-phosphotyrosine antibodies. Differences in immunoprecipitation efficiency and loading were monitored by reprobing stripped membranes with anti-TβR-II antibodies.

### Recombinant GST-Smad3 phosphorylation assay

The phosphorylation of recombinant Smad3 by activated TβR complexes was performed essentially as described previously [45]. Briefly, quiescent NMuMG cells were pretreated with 10 μmol/l of either PP2 or SU6656 (EMD Biosciences, La Jolla, CA, USA) for 1 hour at 37°C, and subsequently were stimulated with TGF-β_1 _(5 ng/ml) for 30 min at 37°C. Cytokine stimulations were terminated by washing the cells twice in ice-cold PBS, which then were lysed and solubilized on ice in buffer H/1% Triton X-100. The resulting cell extracts (1 mg/tube) were clarified by microcentrifugation, and subsequently were immunoprecipitated with anti-TβR-II antibodies for 2 hours at 4°C. TβR immunocomplexes were recovered by brief centrifugation and subsequently were washed twice in PBS. TβR phosphotransferase activity against recombinant GST-Smad3 was measured for 30 min at 30°C in a final reaction volume of 40 μl consisting of 30 μl of TGF-β receptor complexes, 5 mg GST-Smad3, and 10 μl of 4X assay buffer [43]. Phosphotransferase reactions were stopped by the addition of 4X sample buffer and were boiled for 5 min before their fractionation through 10% SDS-PAGE. Fractionated proteins were immobilized electrophoretically to nitrocellulose membranes, which subsequently were probed with anti-phospho-Smad2/3 antibodies to visualize phosphorylated GST-Smad3. Differences in immunoprecipitation efficiency and loading were monitored by reprobing stripped membranes with anti-TβR-II antibodies.

## Results

### TGF-β_1 _mediated EMT increases cell surface α_v_β_3 _integrin expression and β_3 _integrin:TβR-II complex formation in NMuMG cells

**Figure 1:**
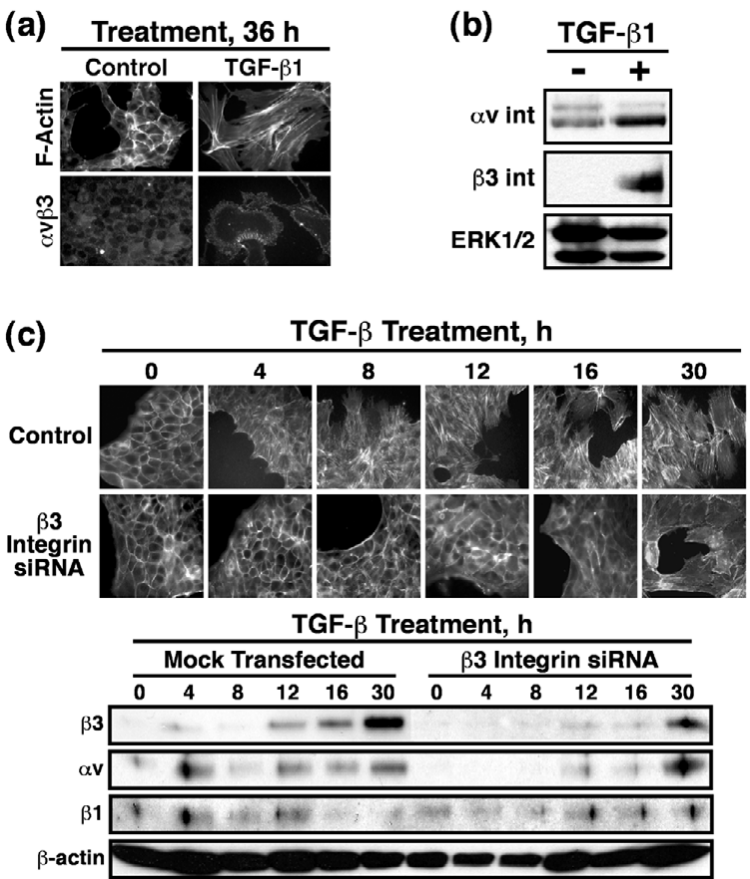
TGF-β_1 _mediated EMT increases cell surface α_v_β_3 _integrin expression in NMuMG cells. NMuMG cells were incubated in the absence or presence of TGF-β_1 _(5 ng/ml) for 0–36 hours and assayed for EMT. **(a) **Alterations in actin cytoskeletal architecture were visualized by direct rhodamine-phalloidin immunofluorescence and α_v_β_3 _integrin by indirect immunofluorescence using anti-α_v_β_3 _integrin (LM609) antibodies. Shown are representative images from a single experiment that was repeated twice with identical results. **(b) **Detergent-solubilized whole cell extracts (50 μg/lane) were prepared to analyze expression of the α_v _and β_3 _integrin subunits by immunoblotting. Differences in protein loading were monitored by reprobing striped blots with anti-ERK1/2 antibodies because, unlike the increase in β-actin expression observed (data not shown) ERK1/2 expression was unaltered in NMuMG cells in response to TGF-β_1 _treatment. **(c) **β_3 _Integrin deficient NMuMG cells were generated by siRNA transfection. The effects of β_3 _integrin deficiency on TGF-β mediated EMT was visualized by direct FITC-phalloidin immunofluorescence. Shown are representative images from a single experiment that was repeated twice with identical results. Detergent-solubilized whole cell extracts (50 μg/lane) were prepared to analyze expression of the α_v_, β_3_, and β_1 _integrin subunits by immunoblotting. Differences in protein loading were monitored by reprobing striped blots with anti-β-actin antibodies. EMT, epithelial-mesenchymal transitions; ERK, extracellular signal-regulated kinase; siRNA, small interfering RNA; TGF, transforming growth factor.

Alterations in cell microenvironments and stromal compartments play critical roles in regulating the progression of tumors from indolent to aggressive states [46,47]. Differential integrin expression [27] and dysregulated TGF-β signaling [48] both contribute to the remodeling of cancer cell microenvironments, particularly those of the breast. Because integrins regulate MEC response to TGF-β [32], we hypothesized that differential integrin expression induced by TGF-β would contribute to its regulation of MEC proliferation and EMT. To test this hypothesis, we stimulated normal mouse mammary epithelial (NMuMG) cells with TGF-β_1 _and monitored changes in actin stress fiber formation by immunofluorescence. As we have shown previously [38], resting NMuMG cells exhibited typical cuboidal epithelial cell morphology characterized by strong cortical acting staining (Figure 1a). When stimulated with TGF-β_1_, these cells underwent EMT and exhibited significant formation of actin stress fibers that emanated from focal adhesions (Figure 1a).

Because α_v_β_3 _integrin expression is elevated in breast cancers and enhances cancer cell invasion [27,30,31], we monitored the ability of TGF-β to alter α_v_β_3 _integrin expression in NMuMG cells. Immunofluorescence analysis showed that TGF-β_1 _treatment of NMuMG cells significantly induced their cell surface expression of the α_v_β_3 _integrin (Figure 1a). Western blot analysis confirmed the upregulation of α_v_β_3 _integrin expression upon TGF-β_1 _mediated EMT (Figure 1b). The association of upregulated α_v_β_3 _integrin expression with TGF-β stimulation of EMT suggested that α_v_β_3 _integrins may play an essential role in facilitating EMT stimulated by TGF-β. We tested this hypothesis by transiently transfecting NMuMG cells with siRNAs directed against β_3 _integrin and subsequently monitored their ability to undergo EMT in response to TGF-β. Figure 1c shows that β_3 _integrin deficiency abolished the ability of TGF-β to stimulate EMT in NMuMG cells. More importantly, NMuMG cells transfected with β_3 _integrin siRNAs only exhibited rudimentary EMT characteristics coordinate with the expression of α_v_β_3 _integrins, which exhibit significantly delayed expression in response to TGF-β (Figure 1c). Moreover, β_3 _integrin deficiency failed to alter NMuMG cell expression of β_1 _integrins (Figure 1c), indicating that signals arising from β_1 _integrin were insufficient in mediating EMT by TGF-β.

Collectively, these findings show that TGF-β induces EMT in NMuMG cells, and that upregulated expression of α_v_β_3 _integrin is essential for this process to occur. The findings also raise the possibility that β_3 _integrins may play a direct role in regulating various MEC responses to TGF-β_3_.

**Figure 2:**
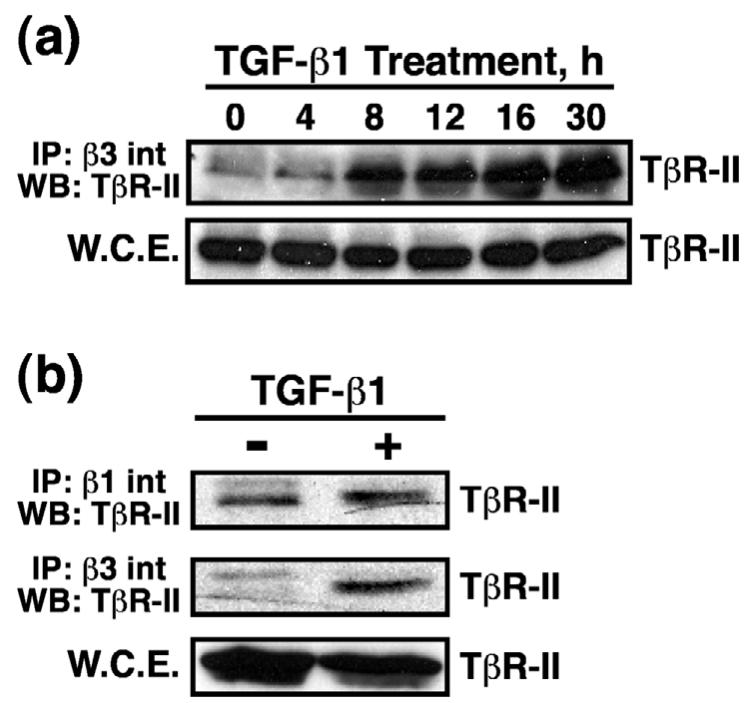
TGF-β_1 _mediated EMT induces β_3 _integrin:TβR-II complex formation on the cell surface of NMuMG cells. NMuMG cells were incubated in the absence or presence of TGF-β_1 _(5 ng/ml) for 0–30 hours to induce EMT. Detergent-solubilized whole cell extracts (1 mg/tube) were immunoprecipitated with **(a,b) **anti-β_3 _or **(b) **anti-β_1 _integrin antibodies, and subsequently immunoblotted with anti-TβR-II antibodies. Shown are representative immunoblots from a single experiment that was repeated twice with identical results. EMT, epithelial-mesenchymal transitions; TβR; TGF receptor; TGF, transforming growth factor; WCE, whole cell extracts.

Integrins regulate growth factor signaling in part via formation of integrin:RTK complexes [23,24]. Based on our findings presented in Figure 1, we hypothesized that β_3 _integrins interact physically with cell surface TβRs. In testing this hypothesis, NMuMG cells were stimulated with TGF-β for various periods of time, whereupon whole cell extracts were prepared and immunoprecipitated with anti-β_3 _integrin antibodies. Probing the resulting immunocomplexes with anti-TβR-II antibodies showed that β_3 _integrin did indeed interact with TβR-II (Figure 2a), and that EMT was necessary to induce this event. In addition, detergent-solubilized whole cell extracts prepared from control and TGF-β stimulated NMuMG cells were immunoprecipitated with antibodies against either β_1 _or β_3 _integrins, and the resulting immunocomplexes were probed for TβR-II. Figure 2b shows that β_1 _integrins interacted constitutively with TβR-II, whereas EMT was again required to promote the association of β_3 _integrin with TβR-II. Collectively, these findings indicate that the formation of β_3 _integrin:TβR-II complexes is an EMT-specific phenomena that may play an important role in regulating TGF-β stimulation of EMT in NMuMG cells.

### β_3 _Integrin overexpression in NMuMG cells

**Figure 3:**
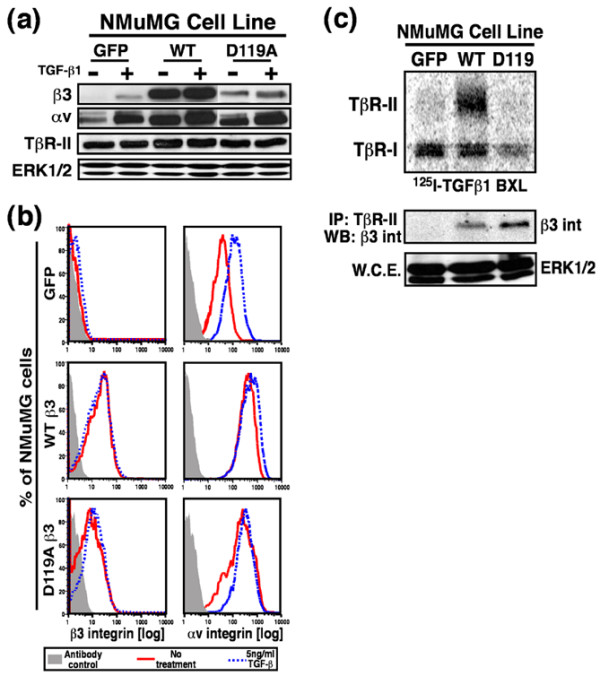
β_3 _Integrin overexpression in NMuMG cells. NMuMG cells were engineered by bi-cistronic retroviral transduction to stably express GFP alone (control), WT β_3 _integrin (WT) and GFP, or its inactive mutant D119A and GFP. **(a) **Detergent-solubilized whole cell extracts (50 μg/lane) were prepared to analyze expression of the α_v _and β_3 _integrin subunits, and of TβR-II. Differences in protein loading were monitored by immunoblotting ERK1/2, whose expression in NMuMG cells was not altered by TGF-β treatment. **(b) **The percentage of NMuMG cells expressing human recombinant β_3 _integrin or endogenous α_v _integrin was determined by FACS analysis of cells incubated with either anti-human β_3 _integrin or anti-mouse α_v _integrin antibodies. Histograms depict β_3 _and α_v _expression only in GFP-positive NMuMG cells and are from a representative experiment that was repeated twice with similar results. **(c) **Iodinated TGF-β_1 _was bound and chemically cross-linked to control (GFP), WT, and D119A β_3 _integrin expressing cells. Cytokine:receptor complexes were isolated by immunoprecipitation with anti-TβR-II antibodies, fractionated through 7.5% SDS-PAGE gels, and immobilized electrophoretically to nitrocellulose membranes. The upper panel shows a representative phosphor image of iodinated TGF-β_1 _bound to TβR-I and TβR-II. The middle panel shows that β_3 _integrin was present in isolated iodinated TGF-β_1_:receptor complexes as detected by anti-β_3 _integrin Western blot analysis of anti-TβR-II immunocomplexes. Differences in protein loading were monitored by probing detergent-solubilized whole cell extracts (50 μg/lane) with anti-ERK1/2 antibodies (bottom panel). BXL, binding and cross-linking; ERK, extracellular signal-regulated kinase; GFP, green fluorescent protein; WT, wild type; TβR, TGF receptor; TGF, transforming growth factor; WCE, whole cell extracts.

To further explore the role of β_3 _integrin in regulating MEC function and TGF-β biology, we used bi-cistronic retroviral transduction to stably express β_3 _integrin or its inactive counterpart D119A-β_3 _integrin [34] in NMuMG cells. The D119A-β_3 _integrin contains a point mutation in the conserved RGD integrin binding motif and therefore fails to bind RGD-containing ligands, such as fibronectin, vitronectin, and osteopontin [49,50]. Recombinant β_3 _integrin expression in NMuMG cells was confirmed by immunoblotting whole cell extracts from basal or TGF-β_1 _treated cells (Figure 3a). As observed previously, TGF-β stimulation of EMT in NMuMG cells induced their expression of α_v _integrin (Figures 1 and 3a). Interestingly, WT β_3 _integrin expression induced a dramatic compensatory upregulation of α_v _integrin expression in NMuMG cells (Figure 3a). This β_3 _integrin response, which was independent of TGF-β administration, was mimicked moderately by expression of its inactive D119A counterpart (Figure 3a). Although D119A β_3 _integrin is considered to be functionally inactive, its heterologous expression in cells can elicit partial biologic activity in part through its ability to bind non-RGD-containing ligands, such as plasminogen [51]. Thus, these findings suggest that signals arising from activated β_3 _integrins induce α_v _integrin expression in MECs.

To confirm that recombinant β_3 _integrins were expressed on the cell surface of NMuMG cells, control or TGF-β_1 _treated cells were incubated with PE-conjugated anti-human β_3 _integrin antibodies, and subsequently analyzed by FACS analysis. As expected, retrovirally infected NMuMG cells expressed WT and D119A β_3 _integrins on their cell surfaces (Fig. 3b, left). In addition, β_3 _integrin expressing cells were also incubated with PE-conjugated anti-mouse α_v _integrin antibodies, which confirmed the compensatory upregulation of α_v _integrins and showed their ability to form functional complexes with cell surface β_3 _integrin (Figure 3b, right).

Finally, iodinated TGF-β_1 _radioligand binding and cross-linking assays were performed to confirm that β_3 _integrins did indeed interact with TβR-II at the cell surface. As shown in Figure 3c, WT and D119A β_3 _integrins both interacted with TβR-II at the cell surface, indicating that formation of this complex is independent of β_3 _integrin activation. Quite surprisingly, WT β_3 _integrin expression, but not that of D119A, increased cell surface expression of TβR-II (Figure 3c). Indeed, the ratio of TβR-II:TβR-I detected at the cell surface in WT β_3 _integrin expressing NMuMG cells (1.31 ± 0.14; *n *= 4) was significantly higher (*P *= 0.009, Student's t-test) than those observed in their GFP β_3 _integrin (0.76 ± 0.05; *n *= 4) and D119A β_3 _integrin (0.72 ± 0.31; *n *= 4) expressing counterparts. Interestingly, semiquantitative real-time PCR analyses failed to detect differences in TβR-II transcript expression between these individual NMuMG cell lines (data not shown), suggesting that signals arising from β_3 _integrins alter TβR-II translocation to and/or internalization from the cell surface of MECs.

Collectively, these findings show that retrovirally expressed human β_3 _integrins were translocated to the cell surface of NMuMG cells, where they formed active heterodimers with α_v _integrin, and more importantly they interacted physically with TβR-II at the plasma membrane. In addition, these findings and those presented in Figures 1 and 2 illustrate a unique and essential role for β_3 _integrin in promoting EMT, particularly that mediated by TGF-β, and suggest that the formation of β_3 _integrin:TβR-II complexes may alter the response of MECs to TGF-β by regulating its intracellular signaling systems.

### β_3 _Integrin expression enhances MAPK activation by TGF-β in NMuMG cells

**Figure 4:**
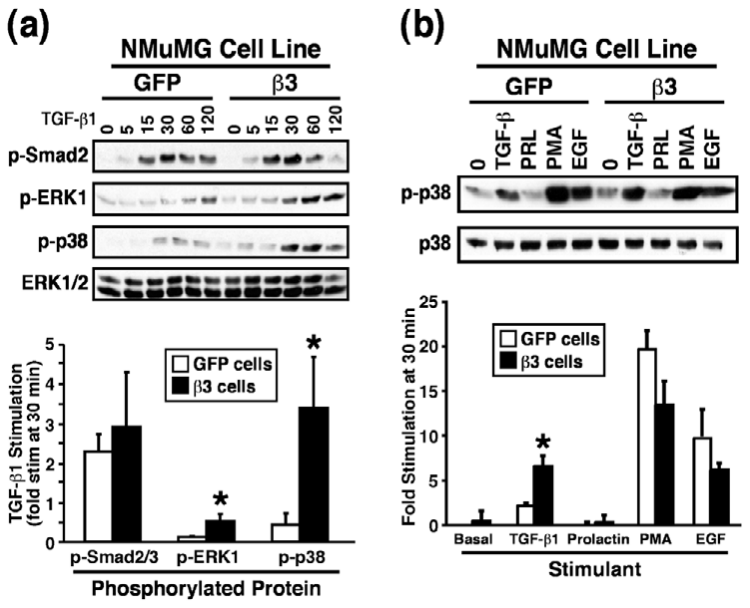
β_3 _Integrin enhances MAPK activation by TGF-β in NMuMG cells. **(a) **Control or β_3 _integrin expressing NMuMG cells were serum starved for 4 hours prior to TGF-β_1 _stimulation (5 ng/ml) for 0–120 min. Afterward, the activation status of Smad2, ERK1/2, and p38 MAPK was determined by immunoblot analysis using phospho-specific antibodies. Reprobing stripped membranes with anti-ERK1/2 antibodies monitored differences in protein loading. **(b) **Control or β_3 _integrin-expressing NMuMG cells were serum starved for 4 hours before stimulation with TGF-β_1 _(5 ng/ml), prolactin (100 ng/ml), PMA (10 ng/ml), or EGF (100 ng/ml) for 30 min. Afterward, the activation status of p38 MAPK was determined by immunoblot analysis using phospho-specific antibodies. Reprobing stripped membranes with anti-pan p38 MAPK antibodies was used to monitor differences in protein loading. Accompanying graphs depict the mean (± standard error) fold increase of p38 MAPK stimulation observed in three independent experiments (*P < 0.05). EGF, epidermal growth factor; ERK, extracellular signal-regulated kinase; MAPK, mitogen-activated protein kinase; PMA, 4α-phorbol 12-myristate 13-acetate; TGF, transforming growth factor.

The coupling of integrins to RTKs often is associated with activation of MAPKs [23,52]. This is noteworthy because the activation of MAPKs is essential for the ability of TGF-β to promote EMT [2,15,16]. We therefore determined whether β_3 _integrin expression altered the ability of TGF-β to activate MAPKs in NMuMG cells. Although expression of β_3 _integrin in NMuMG cells had negligible effects on TGF-β stimulated Smad2 phosphorylation (Figure 4a) and nuclear translocation (data not shown), its expression significantly enhanced the activation of ERK1/2 and p38 MAPK by TGF-β (Figure 4a). Integrin expression can elicit dramatic changes in cell adhesion and morphology [23], potentially resulting in global alterations in intracellular signaling systems. To determine the specificity of β_3 _integrin expression to TGF-β signaling, we monitored the activation status of p38 MAPK in control and β_3 _integrin expressing NMuMG cells before and after their stimulation with TGF-β_1_, prolactin, PMA, or EGF. Figure 4b shows that β_3 _integrin expression selectively regulated the coupling of TGF-β, but not that of prolactin, PMA, or EGF, to p38 MAPK activation in NMuMG cells. Thus, these findings implicate β_3 _integrins as unique mediators of TGF-β signaling in MECs.

### β_3 _Integrin expression blocked TGF-β stimulated growth arrest but enhanced TGF-β mediated invasion and EMT in NMuMG cells

**Figure 5:**
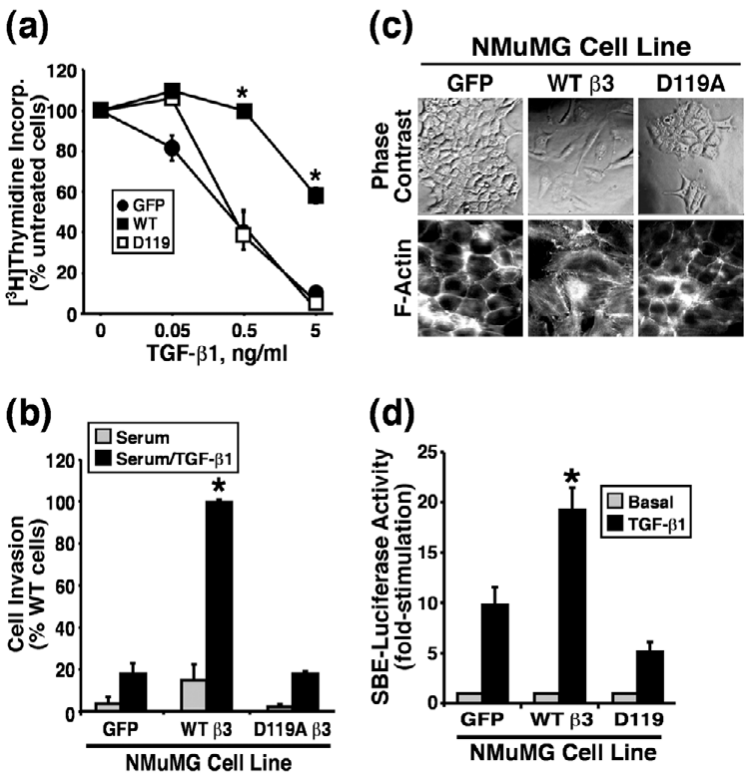
β_3 _Integrin expression blocks TGF-β stimulated growth arrest but enhances TGF-β stimulated invasion and EMT in NMuMG cells. **(a) **Control (GFP), WT, or D119A β_3 _integrin expressing NMuMG cells were stimulated with increasing concentrations of TGF-β_1 _for 48 hours. Cellular DNA was radiolabeled with [^3^H]thymidine and quantified by scintillation counting. Data are the means (± standard error) of three independent experiments presented as the percentage of [^3^H]thymidine incorporation normalized to untreated cells. **(b) **Control (GFP) and β_3 _integrin expressing NMuMG cells were allowed to invade through Matrigel matrices in the absence or presence of TGF-β_1 _(5 ng/ml) for 36 hours. Values are the mean (± standard error) of three independent experiments presented as the percentage invasion relative to TGF-β stimulated β_3 _integrin expressing NMuMG cells. **(c) **The cell morphology of control (GFP), WT, and D119A β_3 _integrin expressing NMuMG cells was monitored by phase contrast microscopy, and alterations in actin cytoskeletal architecture was visualized by direct rhodamine-phalloidin immunofluorescence. Shown are representative images from a single experiment that was repeated twice with identical results. **(d) **Control (GFP), WT, or D119A β_3 _integrin expressing NMuMG cells were transiently transfected with pSBE-luciferase and pCMV-β-gal cDNAs, and subsequently were stimulated with TGF-β_1 _(5 ng/ml) for 24 hours. Afterward, luciferase and β-gal activities contained in detergent-solubilized cell extracts were measured. Values are the mean (± standard error) luciferase activities observed in three independent experiments normalized to maximal reporter gene expression measured in unstimulated cells. EMT, epithelial-mesenchymal transitions; GFP, green fluorescent protein; TGF, transforming growth factor; WT, wild type; SBE, Smad binding element.

Based upon the ability of β_3 _integrins to enhance MAPK activation by TGF-β, we next sought to determine whether β_3 _integrins could alter the ability of TGF-β to regulate MEC cell proliferation, invasion, and EMT. We first measured the effects of β_3 _integrin expression on the rate of DNA synthesis in resting NMuMG cells. As compared with GFP expressing NMuMG cells, those expressing WT β_3 _integrin exhibited significantly increased rates of DNA synthesis (by 78 ± 0.12%; *n *= 3; *P *= 0.002), whereas those expressing D119A β_3 _integrin exhibited significantly retarded rates of DNA synthesis (by 68 ± 4.0%; *n *= 3; *P *= 0.00007). Despite the relative growth rate differences observed between these β_3 _integrin expressing NMuMG cell derivatives, the response of GFP and D119A expressing NMuMG cells to TGF-β mediated cytostasis was surprisingly similar (Figure 5a). In contrast, WT β_3 _integrin expression significantly reduced the ability of TGF-β to induce growth arrest in NMuMG cells (Figure 5a), indicating that β_3 _integrin expression significantly reduces cytostasis mediated by TGF-β, thereby circumventing its tumor suppressor function in MECs.

We also determined whether β_3 _integrins are capable of enhancing the tumor promoting functions of TGF-β. In doing so, we compared the ability of β_3 _integrin expressing NMuMG cell lines to invade through synthetic basement membranes. As shown in Figure 5b, GFP expressing NMuMG cells exhibited minimal invasion through Matrigel matrices in response to serum stimulation, a response that was enhanced insignificantly by TGF-β treatment. Similar to its inability to alter growth regulation by TGF-β, expression of the D119A β_3 _integrin in NMuMG cells failed to affect their TGF-β regulated invasiveness (Figure 5b). In contrast, NMuMG cells expressing β_3 _integrin exhibited a trend toward increased invasiveness to serum as compared with control cells (Figure 5b); however, when stimulated with TGF-β_1 _these cells exhibited significantly enhanced invasion through synthetic basement membranes (Figure 5b). Because increased invasiveness is often associated with EMT [11], we examined the morphology and actin cytoskeleton of NMuMG cells expressing β_3 _integrins. Consistent with our previous results, only those NMuMG cells that expressed WT β_3 _integrin acquired an elongated, fibroblast-like morphology (Figure 5c), complete with the formation of actin stress fibers (Figure 5c). Taken together, these findings suggest that although both WT and D119A β_3 _integrins can form complexes with TβR-II at the cell surface, only functional β_3 _integrins are capable of inducing EMT and invasion in NMuMG cells.

Lastly, we monitored changes in TGF-β stimulated gene expression by measuring differences in luciferase expression driven by the synthetic SBE (Smad binding element) promoter. Figure 5d shows that only WT β_3 _integrin expression significantly enhanced TGF-β stimulated gene expression as compared with control cells or those expressing its inactive β_3 _integrin counterpart.

Collectively, these findings indicate that, unlike its nonfunctional counterparts, expression of WT β_3 _integrin in MECs diminished TGF-β mediated growth arrest, enhanced TGF-β mediated invasion and EMT, and augmented TGF-β mediated gene expression. Moreover, our results suggest that β_3 _integrin expression translates TGF-β from an inhibitor of MEC cell growth to a stimulator of their invasion and EMT.

### Dual receptor activation induces Src-mediated TβR-II tyrosine phosphorylation

**Figure 6:**
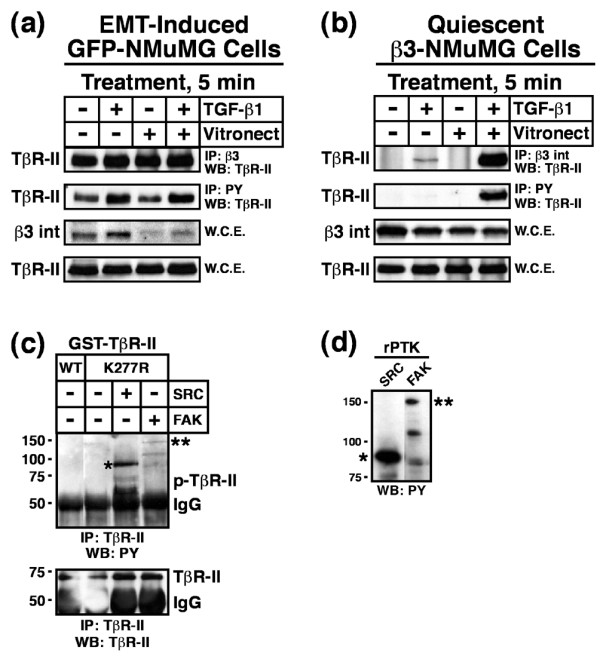
Dual receptor activation induces Src mediated TβR-II tyrosine phosphorylation. **(a) **GFP-expressing NMuMG cells were stimulated with TGF-β_1 _(5 ng/ml) for 36 hours to induce β_3 _integrin expression and EMT. Afterward, the cells were dissociated and re-plated either onto plastic or vitronectin-coated wells in the absence or presence of TGF-β_1 _(5 ng/ml) for 5 min. Afterward, detergent-solubilized whole cell extracts were prepared (1 mg/tube) and subsequently immunoprecipitated with anti-phosphotyrosine or anti-β_3 _integrin antibodies as indicated. The presence of TβR-II in precipitated immunocomplexes was determined by Western blotting with anti-TβR-II antibodies. Differences in protein loading were monitored by immunoblotting whole cell extracts (50 mg/lane) for β_3 _integrin and TβR-II. Shown are representative immunoblots from a single experiment that was repeated twice with similar results. **(b) **Resting WT β_3 _integrin-expressing NMuMG cells were dissociated and re-plated as above. Afterward, TβR-II tyrosine phosphorylation and complex formation with β_3 _integrin was determined by immunoblotting as above. Shown are representative immunoblots from a single experiment that was repeated three times with identical results. **(c) **Active recombinant Src (1 unit/reaction) or FAK (0.2 μg/reaction) kinases were added to protein kinase reaction mixtures containing 1 μg/tube of either kinase active (WT) or inactive (K277R) GST-TβR-II. Phosphorylation reactions were stopped after 30 min and the phosphorylation status of TβR-II was determined by immunoprecipitation of diluted protein kinase reaction mixtures with anti-TβR-II antibodies. Afterward, immobilized immunocomplexes were probed sequentially with anti-phosphotyrosine antibodies, followed by anti-TβR-II antibodies as indicated (*autophosphorylated recombinant Src; **autophosphorylated recombinant FAK). Data are from a representative experiment that was repeated three times with similar results. **(d) **Recombinant Src and FAK were incubated in protein kinase assay buffer for 30 min at 30°C. The data show the autophosphorylation on tyrosine residues of recombinant Src (*) and FAK (**) as determined by immunoblotting with anti-phosphotyrosine antibodies. Data are from a representative experiment that was repeated at least three times with similar results. EMT, epithelial-mesenchymal transitions; FAK, focal adhesion kinase; GST, glutathione *S*-transferase; TβR, TGF-β receptor; TGF, transforming growth factor; WCE, whole cell extracts; WT, wild type.

Our findings thus far show that β_3 _integrin and TβR-II interact upon TGF-β stimulation, β_3 _integrin expression is essential for EMT stimulated by TGF-β, and β_3 _integrin expression influences the MEC response to TGF-β. To further explore the consequences of β_3 _integrin:TβR-II complex formation, GFP expressing NMuMG cells were incubated in the absence or presence of TGF-β for 36 hours, and subsequently were held in suspension before re-plating onto plastic or vitronectin-coated wells in the absence or presence of TGF-β_1 _for 5 min. Afterward, the phosphorylation status of TβR-II and its ability to form complexes with β_3 _integrin were monitored by co-immunoprecipitation analyses. Figure 6a shows that following TGF-β induced EMT, TβR-II was constitutively tyrosine phosphorylated and associated with β_3 _integrin. Because chronic TGF-β stimulation of NMuMG cells induces β_3 _integrin expression (Figure 1) and complex formation with TβR-II (Figure 2), it was difficult to assess the role of TGF-β receptor and integrin stimulation in the formation of β_3 _integrin:TβR-II complexes in NMuMG cells. To circumvent this difficulty, resting WT β_3 _integrin expressing NMuMG cells were dissociated and subsequently replated and stimulated for 5 min, as above. Figure 6b shows that TβR-II only interacted with β_3 _integrins upon TGF-β_1 _stimulation and, more importantly, that this response was enhanced significantly upon β_3 _integrin adhesion. In addition, only upon dual receptor activation (i.e. β_3 _integrin and TβRs) was TβR-II phosphorylated on tyrosine residues (Figure 6b). Thus, β_3 _integrin regulates TGF-β signaling by targeting TβR-II for tyrosine phosphorylation in a manner reminiscent of integrin actions on RTK signaling [23].

Although integrins lack intrinsic protein tyrosine kinase (PTK) activity, they facilitate tyrosine phosphorylation of target proteins via their recruitment of FAK and Src PTKs to focal adhesions [22]. We therefore hypothesized that FAK or Src are potential PTKs responsible for phosphorylating TβR-II on tyrosine residues in NMuMG cells. We tested this hypothesis by utilizing an *in vitro *PTK assay that measured the ability of purified, active FAK or Src to phosphorylate GST fusion proteins containing catalytically active or inactive (i.e. K277R) versions of the cytoplasmic domain of TβR-II. As shown in Figure 6c, Src phosphorylated the cytoplasmic domain of TβR-II on tyrosine residues. Although active and capable of undergoing autophosphorylation on tyrosine residues (Figure 6d), FAK was unable to phosphorylate TβR-II *in vitro *(Figure 6c), suggesting that Src phosphorylates TβR-II in response to β_3 _integrin expression.

**Figure 7:**
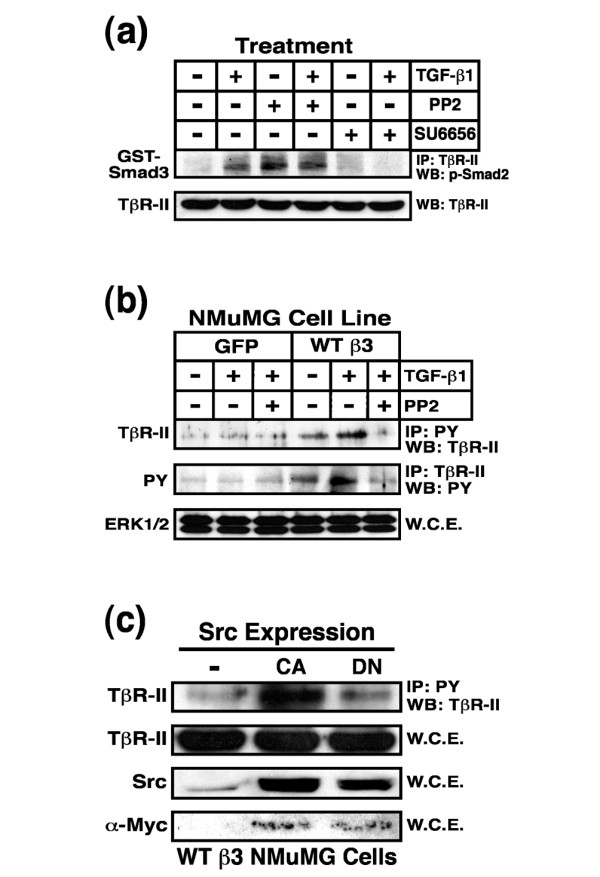
Src inhibition blocks TβR-II tyrosine phosphorylation in NMuMG cells. **(a) **NMuMG cells were pretreated for 60 min with either PP2 (10 μmol/l) or SU6656 (10 μmol/l). Afterward, the cells were stimulated with TGF-β_1 _(5 ng/ml) for 20 min, and subsequently were lysed and immunoprecipitated (1 mg/tube) with anti-TβR-II antibodies. The resulting immunocomplexes were used to phosphorylate recombinant GST-Smad3, which was visualized by immunoblotting with anti-phospho-Smad2 antibodies. Data are from a representative experiment that was repeated three times with similar results. **(b) **Control or β_3 _integrin expressing NMuMG cells were stimulated with TGF-β_1 _(5 ng/ml) in the absence or presence the Src inhibitor PP2 (10 μmol/l), as indicated. Afterward, detergent-solubilized whole cell extracts were prepared, immunoprecipitated, and subjected to Western blot analysis either with anti-phosphotyrosine (2 mg WCE/tube) or anti-TβR-II (1 mg WCE/tube) antibodies as indicated. Differences in protein loading were monitored by probing detergent-solubilized whole cell extracts (50 μg/lane) with anti-ERK1/2 antibodies (bottom panel). **(c) **WT β_3 _integrin expressing NMuMG cells were transiently transfected with cDNAs encoding either constitutively active (CA) or dominant negative (DN) Src. Afterward, detergent solubilized whole cell extracts were prepared, immunoprecipitated with anti-phosphotyrosine (1 mg WCE/tube), and subjected to WB analysis with anti-TβR-II antibodies. Total Src expression was monitored by probing detergent solubilized whole cell extracts (50 μg/lane) with anti-Src antibodies, whereas exogenous Src expression was confirmed by reprobing stripped membranes with anti-Myc antibodies. Differences in protein loading were monitored by probing stripped membranes with anti-TβR-II antibodies. ERK, extracellular signal-regulated kinase; GST, glutathione *S*-transferase; IP, immunoprecipitated; PY, phosphotyrosine; TβR, TGF-β receptor; TGF, transforming growth factor; WB, Western blot; WCE, whole cell extracts; WT, wild type.

To further examine the functional link between Src and TβR-II, we attempted to inhibit Src activity or function in NMuMG cells and subsequently monitor the consequences of these manipulations on TβR-II tyrosine phosphorylation. Recent work conducted by Maeda and coworkers [53] showed that the broad spectrum protein kinase inhibitor PP1 antagonizes TGF-β signaling by inhibiting TβR-I and TβR-II protein kinase activity. As such, we monitored the effects of two Src inhibitors, PP2 and SU6656, on the ability of immunoprecipitated TGF-β receptor complexes to phosphorylate recombinant GST-Smad3 *in vitro*. As expected, TGF-β stimulation greatly enhanced the phosphorylation of recombinant Smad3 (Figure 7a), a reaction that was reduced markedly by SU6656 administration (Figure 7a). In contrast, PP2 treatment failed to inhibit TβR activity and instead appeared to elicit agonist independent receptor activation (Figure 7a). Based on its inactivity against TβRs, PP2 appeared to be a suitable pharmacological agent to inhibit Src activity in MECs. Accordingly, β_3 _integrin-expressing NMuMG cells treated with PP2 abrogated the tyrosine phosphorylation of TβR-II in TGF-β stimulated NMuMG cells (Figure 7b), indicating that Src activity does indeed mediate tyrosine phosphorylation of TβR-II in β_3 _integrin expressing NMuMG cells. Along these lines, transient expression of constitutively active Src in β_3 _integrin expressing NMuMG cells resulted in significantly elevated tyrosine phosphorylation of TβR-II, an event that was not recapitulated by expression of dominant negative Src (Figure 7c). Taken together, these findings indicate that β_3 _integrin-mediated recruitment of Src facilitates the tyrosine phosphorylation of TβR-II in MECs, and that this phosphorylation event may act to enhance their proliferation, invasion, and EMT by augmenting the ability of TGF-β to activate MAPKs.

### Src inhibition blocks β_3 _integrin and TGF-β mediated MAPK activation, EMT, and invasion in NMuMG cells

**Figure 8:**
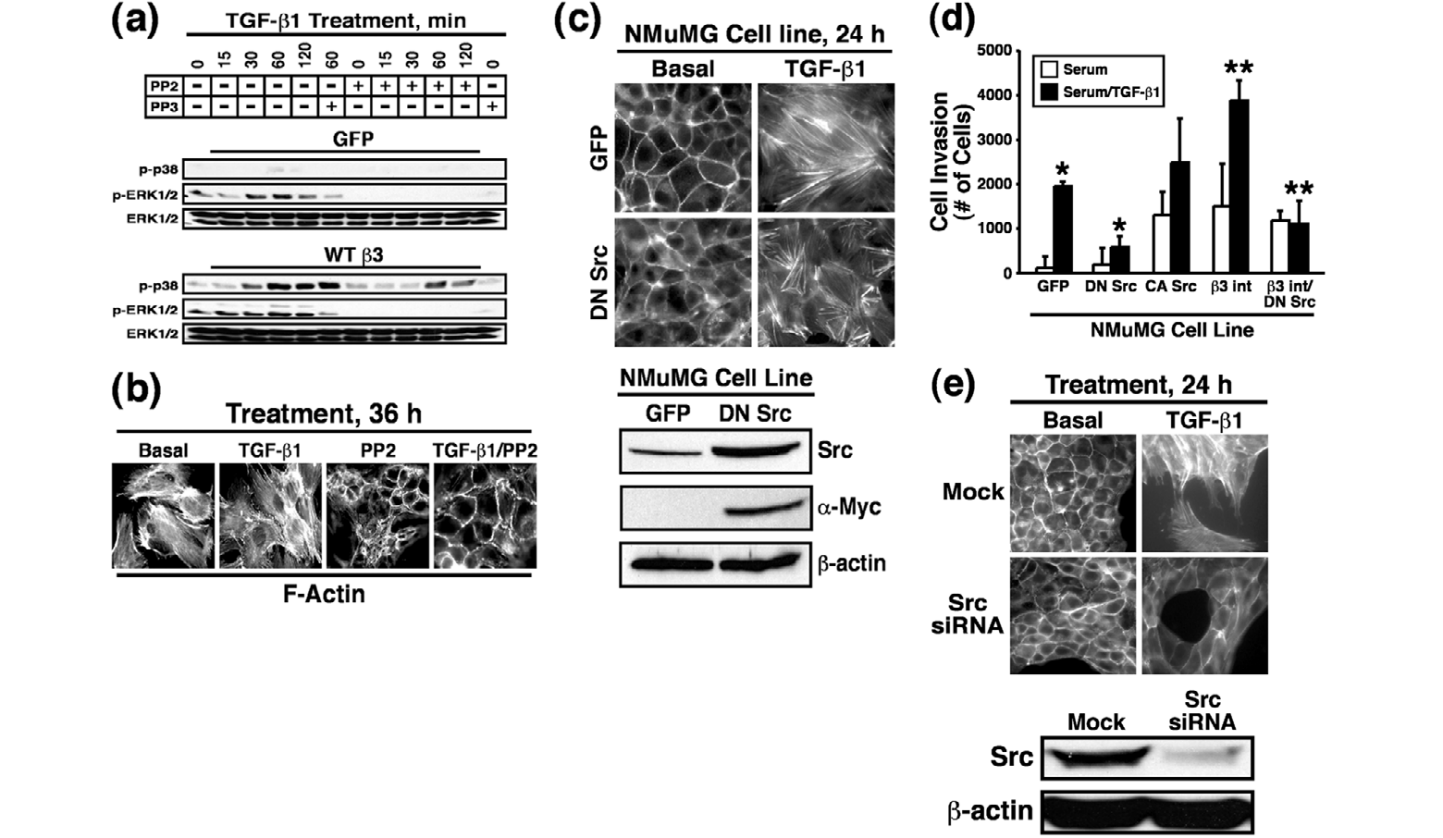
Src inhibition blocks β_3 _integrin and TGF-β mediated MAPK activation, EMT, and invasion in NMuMG cells. **(a) **Control or β_3 _integrin expressing NMuMG cells were serum starved for 4 hours before TGF-β_1 _(5 ng/ml) stimulation in the absence or presence of either PP2 (10 μmol/l) or its inactive counterpart PP3 (10 μmol/l). Afterward, the activation status of ERK1/2 and p38 MAPK was determined by immunoblot analysis using phospho-specific antibodies. Re-probing stripped membranes with anti-ERK1/2 antibodies monitored differences in protein loading. Data are from a representative experiment that was repeated twice with similar results. **(b) **β_3 _Integrin expressing NMuMG cells were stimulated with TGF-β_1 _(5 ng/ml) for 36 hours in the absence or presence of PP2 (10 μmol/l). Afterward, alterations in actin cytoskeletal architecture were visualized by direct rhodamine-phalloidin immunofluorescence. Shown are representative images from a single experiment that was repeated twice with identical results. **(c) **Control (GFP) or DN Src-expressing NMuMG cells were stimulated with TGF-β_1 _(5 ng/ml) for 24 hours. Afterward, alterations in actin cytoskeletal architecture were visualized by direct rhodamine-phalloidin immunofluorescence. Total Src expression was monitored by probing detergent-solubilized whole cell extracts (50 μg/lane) with anti-Src antibodies, whereas exogenous Src expression was visualized using anti-Myc antibodies. Immunoblotting stripped membranes with anti-β-actin antibodies served to monitor differences in protein loading. Shown are representative images from a single experiment that was repeated twice with identical results. **(d) **Control (GFP), DN Src, CA Src, β_3 _integrin, and β_3 _integrin/DN Src expressing NMuMG cells were allowed to invade through Matrigel matrices in the absence or presence of TGF-β_1 _(5 ng/ml) for 36 hours. Values are the mean (± standard error) of three independent experiments presented as the number of cells invaded per well. **(e) **Src-deficient NMuMG cells were generated by siRNA transfection. The effects of Src deficiency on TGF-β mediated EMT were visualized by direct FITC-phalloidin immunofluorescence. Shown are representative images from a single experiment that was repeated twice with identical results. Detergent-solubilized whole cell extracts (50 μg/lane) were prepared to monitor Src expression by immunoblotting with anti-Src antibodies. Differences in protein loading were monitored by re-probing striped blots with anti-β-actin antibodies. CA, constitutively active; DN, dominant negative; ERK, extracellular signal-regulated kinase; GFP, green fluorescent protein; MAPK, mitogen-activated protein kinase; TGF, transforming growth factor; WB, Western blot; WT, wild type.

We next explored the functional importance of Src in regulating various MEC responses to TGF-β. To do so, NMuMG cells were pretreated with either PP2 or its inactive counterpart PP3 and subsequently stimulated with TGF-β to activate ERK1/2 and p38 MAPK. As expected, β_3 _integrin expression significantly increased ERK1/2 and p38 MAPK activation following TGF-β_1 _stimulation (Figure 8a). This response to TGF-β was unaffected by PP3 administration, but it was reduced significantly by PP2 treatment of NMuMG cells (Figure 8a). More importantly, PP2 administration completely reverted the EMT phenotype induced by expression of β_3 _integrin in NMuMG cells, and nearly abolished the ability of TGF-β to stimulate EMT in NMuMG cells (Figure 8b). We also engineered NMuMG cells to stably express dominant negative Src and subsequently measured its effect on the ability of TGF-β to induce EMT and invasion in MECs. As expected, GFP expressing NMuMG cells readily underwent EMT when stimulated by TGF-β (Figure 8c). In stark contrast, NMuMG cells expressing dominant negative Src were stunted in their ability to undergo EMT in response to TGF-β (Figure 8c), and instead formed small, disorganized stress fibers that contrasted sharply with those produced in their GFP expressing counterparts.

Proper organization of cell polarity is essential for cancer cell invasion and metastasis [54]. Based on the aberrant EMT response observed in dominant negative Src expressing NMuMG cells, we suspected that their invasion through synthetic basement membranes would also be impaired. Accordingly, dominant negative Src expression significantly inhibited the ability of TGF-β to induce NMuMG cell invasion (Figure 8d). Interestingly, singular expression of either constitutively active Src or β_3 _integrin in NMuMG cells significantly enhanced tonic and TGF-β stimulated cell invasion (Figure 8d). More importantly, introducing dominant negative Src into β_3 _integrin expressing NMuMG cells completely abolished the ability of TGF-β to stimulate cell invasion in MECs (Figure 8d). Thus, activity of both β_3 _integrin and Src is necessary for MEC invasion stimulated by TGF-β.

Finally, the findings above suggest that Src may play an essential role in facilitating EMT stimulated by TGF-β. We tested this hypothesis by transiently transfecting NMuMG cells with siRNAs directed against Src and subsequently monitored their ability to undergo EMT in response to TGF-β. Figure 8e shows that Src deficiency did indeed abolish the ability of TGF-β to stimulate EMT in NMuMG cells, a response reminiscent of that observed in NMuMG cells deficient in β_3 _integrin (Figure 1c). Collectively, these findings indicate that Src expression and activity are essential for the ability of TGF-β to stimulate MAPKs, and to induce EMT and cell invasion in MECs.

### β_3 _Integrin expression increases in malignant human MECs and regulates invasion stimulated by TGF-β

Our findings have thus far solely investigated the relationship between β_3 _integrins and TGF-β in normal MECs. It should be noted that cells undergoing neoplastic transformation, including those of the breast [26,27], exhibit altered integrin expression profiles during tumorigenesis, suggesting that integrins could play a key role in the acquisition of neoplastic phenotypes. The human MCF10A system consists of a series MEC cell lines that share a common ancestry and represent distinct stages of breast cancer development, ranging from normal to highly invasive and metastatic [55]; they also represent a model system for studying the conversion of TGF-β from a tumor suppressor to a tumor promoter [9].

**Figure 9:**
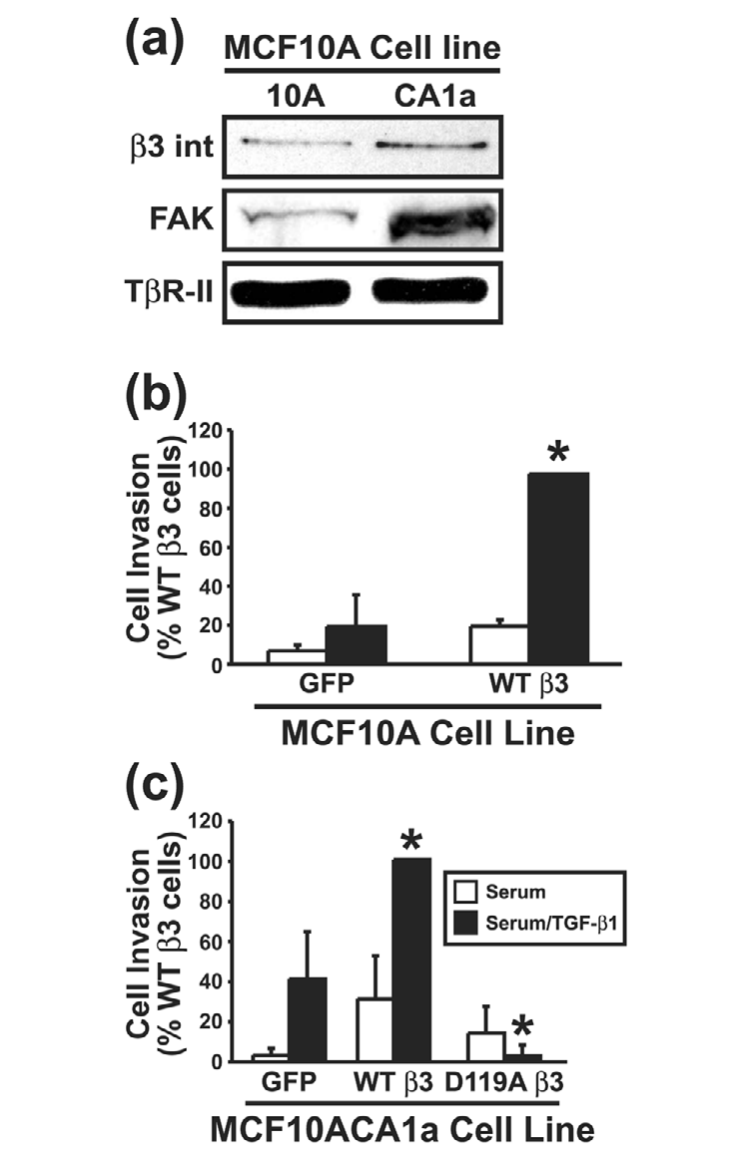
β_3 _Integrin expression increases in malignant human MECs and regulates invasion stimulated by TGF-β. **(a) **Detergent-solubilized whole cell extracts (100 μg/lane) of MCF10A and MCF10CA1a cells were prepared to analyze expression of the β_3 _integrin subunit and FAK by immunoblotting. Differences in protein loading were monitored by probing striped blots with anti-TβR-II antibodies. Control (GFP) and β_3 _integrin expressing **(b) **MCF10A cells or **(c) **MCF10CA1a cells were allowed to invade through Matrigel matrices in the absence or presence of TGF-β_1 _(5 ng/ml) for 36 hours. Values are the mean (± standard error) of three independent experiments presented as the percentage invasion relative to TGF-β stimulated WT β_3 _integrin expressing cells. FAK, focal adhesion kinase; MEC, mammary epithelial cell; TβR, TGF-β receptor; TGF, transforming growth factor; WT, wild type.

Interestingly, breast cancer progression was associated with elevated expression of β_3 _integrin and its effector, FAK, which correlated with the conversion of TGF-β from a suppressor to a promoter of breast cancer progression (Figure 9a). Moreover, exogenous β_3 _integrin expression in normal, poorly invasive MCF10A cells significantly enhanced their invasion through synthetic basement membranes in response to TGF-β (Figure 9b). In contrast, the malignant, metastatic MCF10CA1a cells readily invaded Matrigel matrices when stimulated with TGF-β, a response that was augmented significantly by WT β_3 _integrin expression (Figure 9c). Importantly, D119A β_3 _integrin expression completely abolished the ability of TGF-β to stimulate MCF10ACA1a cell invasion (Figure 9c). Collectively, our findings suggest that mammary tumorigenesis alters β_3 _integrin expression in neoplastic MECs, and that β_3 _integrins may play a crucial role in translating TGF-β from a suppressor to a promoter of breast cancer growth and invasion.

## Discussion

The process of EMT recently has been subject to intense research investigation because of its importance in mediating cancer cell motility, invasion, and metastasis [11,48]. TGF-β is a major regulator of both developmental and pathological EMT, particularly that in diseased MECs, which frequently exhibit decreased cytostasis and increased invasiveness in response to TGF-β [56]. Unfortunately, the molecular mechanisms underlying the conversion of TGF-β from an inhibitor of MEC proliferation to an inducer of their EMT are poorly understood. Because cell microenvironmental changes regulate cancer development and progression, including the acquisition of EMT, and because integrins and TGF-β are major regulators of cell microenvironments, we hypothesized that differential integrin expression induced by TGF-β would contribute to its regulation of MEC proliferation and EMT.

To this end, we now show that β_3 _integrin expression alters the response of MECs to TGF-β, particularly its ability to regulate cell proliferation, invasion, and EMT. Indeed, β_3 _integrin expression appears essential for TGF-β stimulation of EMT (Figure 1c). Moreover, we demonstrate that β_3 _integrin directly couples to the TGF-β signaling system by interacting physically with TβR-II (Figure 2), resulting in enhanced MEC proliferation, invasion, and EMT when stimulated with TGF-β (Figure 5). More importantly, β_3 _integrin promotes Src mediated tyrosine phosphorylation of TβR-II (Figure 6), which we show to be essential for the ability of TGF-β to activate MAPKs (Figure 8) and, consequently, to induce EMT in MECs (Figure 8). More importantly, we show for the first time that induction of β_3 _integrin expression during mammary tumorigenesis correlates with metastasis development, and that antagonizing β_3 _integrin signaling abolishes TGF-β stimulation of breast cancer cell invasion (Figure 9). Collectively, we identified a potentially important mechanism whereby β_3 _integrin expression selectively facilitates TGF-β stimulation of MEC invasion and EMT.

The joint activation of integrin and RTK signaling systems is essential for the positional control of cells. More importantly, cell attachment to the ECM also determines the nature and context of how cells interpret and respond to cytokine and growth factor binding [52]. For instance, the ability of EGF and platelet-derived growth factor receptors to stimulate fibroblast migration requires RTK receptor association with integrins and FAK [24]. Similarly, Scaffidi and coworkers [42] showed that α_v_β_3 _integrin bound TβR-II in lung fibroblasts, thereby enhancing fibroblast proliferation stimulated by TGF-β. Those authors further speculated that the formation of α_v_β_3 _integrin:TβR-II complexes may exacerbate TGF-β mediated wound healing and fibrotic reactions [42], processes reminiscent of the ability of β_3 _integrin:TβR-II complexes to drive TGF-β stimulation of EMT in MECs. Most recently, the adapter protein Dab2, which mediates Smad2/3 activation by TβRs [57], was shown to participate in TGF-β stimulated EMT by interacting with integrins and preventing MEC apoptosis [58]. These findings, together with those presented herein, indicate an important and underappreciated role for integrins in regulating cellular response to TGF-β. Future studies in MECs must determine the relationship between TGF-β stimulated expression of β_3 _integrin and Dab2, as well as β_3 _integrin stimulated TβR-II expression in MECs; whether Dab2 links β_3 _integrin to TβR-II during EMT; and the ability of additional integrins to influence, either positively or negatively, MEC response to TGF-β.

A particularly novel finding of our study was the demonstration that β_3 _integrin induced TβR-II tyrosine phosphorylation via Src kinase. Indeed, we show for the first time that Src activity mediates tyrosine phosphorylation of TβR-II in MECs, leading to TGF-β mediated MAPK activation, and to MEC invasion and EMT. Previous studies established that TβR-II is phosphorylated predominantly on Ser and Thr residues [8,13,59], which, depending on the site of phosphorylation, either augments or attenuates TβR-II protein kinase activity [60]. However, tyrosine phosphorylation of TβR-II is not without precedent. Indeed, Lawler and coworkers [61] showed that TβR-II is a dual-specificity protein kinase that autophosphorylates not only on Ser/Thr residues but also on tyrosine 259, 336, and 424. Moreover, although Phe substitution at these positions significantly reduced TβR-II protein kinase activity, these mutations had little affect on the ability of TGF-β to induce gene expression in lung epithelial cells [61], and as such the role of tyrosines 259, 336, and 424 in mediating TGF-β action remain to be clarified. We too have converted tyrosines 259, 336, and 424 to phenylalanine in all possible combinations (i.e. single, double, and triple mutations), all of which failed to alter Src mediated tyrosine phosphorylation of GST-TβR-II (K277R) *in vitro *(Galliher AJ, Schiemann WP, unpublished data). Sequence analysis of the cytoplasmic domain of TβR-II revealed two possible Src phosphorylation consensus motifs at tyrosines 284 and 470. These two sites were individually mutated to phenylalanines, and preliminary data suggest that Src phosphorylates TβR-II at tyrosine 284 (Galliher AJ, Schiemann WP, unpublished data). Further analysis of this site in mediating TGF-β stimulation of EMT in MECs is currently under investigation.

Activation of MAPKs, particularly that of ERK1/2 [5] and p38 MAPK [2,32], are necessary for TGF-β to stimulate EMT. We also found that TGF-β stimulation of ERK1/2 and p38 MAPK are necessary for induction of EMT in MECs. More importantly, we show for the first time that Src activity is essential for activation of MAPKs by TGF-β and for its stimulation of EMT. Indeed, our results indicate that β_3 _integrin expression and Src activity are sufficient in overcoming TGF-β mediated cytostasis in MECs. Src activity also has been associated with protecting hepatocytes from apoptosis induced by TGF-β [62-64] and with the ability of TGF-β to stimulate ovarian cancer cell invasion [65]. More recently, TGF-β treatment of MECs was shown to induce their expression of RPTPκ, a protein tyrosine phosphatase that activates Src and mediates the antiproliferative and the promigratory effects of TGF-β [66]. These findings, together with those presented herein, implicate Src as an important player operant in dictating the MEC response to TGF-β; they also suggest that Src inhibition, similar to integrin interdiction, may one day be used to enhance the tumor suppressing activities of TGF-β in breast cancer cells.

Finally, based on the fact that phosphotyrosine residues often create binding motifs that couple receptors to various signaling pathways [67], including the MAPK cascade, it is tempting to speculate that Src-mediated tyrosine phosphorylation of TβR-II functions similarly as a receptor docking site to recruit SH2 and/or PTB containing signaling molecules to β_3 _integrin:TβR-II complexes. If correct, then such a mechanism could account for the augmented ability of TGF-β to activate MAPKs in β_3 _integrin expressing MECs, and for the shift in MAPK and Smad2/3 signaling that favored EMT over cytostasis in response to TGF-β. Indeed, the ability of β_3 _integrin to increase Smad2/3 transcriptional activity without altering Smad2/3 phosphorylation suggests that other integrin or MAPK stimulated nuclear factors converge on TGF-β targeted promoters and synergize with Smad2/3 in coordinating gene expression regulated by TGF-β. This notion is wholly consistent with previous work demonstrating the ability of Smad2/3 to synergize with activated TβR complexes in mediating EMT [37,68].

## Conclusion

In summary, we present evidence that the differential expression of β_3 _integrin induced by TGF-β functions to convert this cytokine from an inhibitor of MEC proliferation to a stimulator of their EMT and invasion. More importantly, we show that β_3 _integrin regulates TGF-β signaling by interacting physically with TβR-II and stimulating its phosphorylation on tyrosine residues by Src. The net effect of these events results in enhanced TGF-β stimulation of MAPKs and, consequently, induction of EMT and invasion in MECs. Indeed, our findings suggest that integrin interdiction may prevent tumor development and progression by maintaining, reinforcing, or re-establishing the tumor suppressing activities of TGF-β.

## Abbreviations

ECM = extracellular matrix; EGF = epidermal growth factor; EMT = epithelial-mesenchymal transitions; ERK = extracellular signal-regulated kinase; FACS = fluorescence-activated cell sorter; FAK = focal adhesion kinase; FBS = fetal bovine serum; GFP = green fluorescent protein; GST = glutathione *S*-transferase; IRES = internal ribosomal entry site; MAPK = mitogen-activated protein kinase; MEC = mammary epithelial cell; PBS = phosphate-buffered saline; PCR = polymerase chain reaction; PI3K = phosphoinositol-3 kinase; PMA = 4α-phorbol 12-myristate 13-acetate; pMSCV = plasmid murine stem cell virus; PTK = protein tyrosine kinase; RGD = arginine-glycine-aspartic acid; RTK = receptor tyrosine kinase; siRNA = small interfering RNA; TβR = TGF-β receptor; TGF = transforming growth factor; WT = wild type; YFP = yellow fluorescent protein.

## Competing interests

The authors declare that they have no competing interests.

## Authors' contributions

AJG participated in designing the study, acquiring data, interpreting data, analyzing data, and preparing the manuscript. WPS participated in designing the study, interpreting data, and preparing the manuscript.
